# cFLIP in the molecular regulation of astroglia-driven neuroinflammation in experimental glaucoma

**DOI:** 10.1186/s12974-024-03141-4

**Published:** 2024-06-01

**Authors:** Xiangjun Yang, Qun Zeng, Maide Gözde İnam, Onur İnam, Chyuan-Sheng Lin, Gülgün Tezel

**Affiliations:** 1 Department of Ophthalmology, Vagelos College of Physicians and Surgeons, Columbia University, New York, NY 10032 USA; 2 Department of Pathology & Cell Biology, Vagelos College of Physicians and Surgeons, Columbia University, New York, NY USA

**Keywords:** Astroglia, cFLIP, Cytokine, Glaucoma, Neuroinflammation

## Abstract

**Background:**

Recent experimental studies of neuroinflammation in glaucoma pointed to cFLIP as a molecular switch for cell fate decisions, mainly regulating cell type-specific caspase-8 functions in cell death and inflammation. This study aimed to determine the importance of cFLIP for regulating astroglia-driven neuroinflammation in experimental glaucoma by analyzing the outcomes of astroglia-targeted transgenic deletion of *cFLIP* or *cFLIP*_*L*_.

**Methods:**

Glaucoma was modeled by anterior chamber microbead injections to induce ocular hypertension in mouse lines with or without conditional deletion of *cFLIP* or *cFLIP*_*L*_ in astroglia. Morphological analysis of astroglia responses assessed quantitative parameters in retinal whole mounts immunolabeled for GFAP and inflammatory molecules or assayed for TUNEL. The molecular analysis included 36-plexed immunoassays of the retina and optic nerve cytokines and chemokines, NanoString-based profiling of inflammation-related gene expression, and Western blot analysis of selected proteins in freshly isolated samples of astroglia.

**Results:**

Immunoassays and immunolabeling of retina and optic nerve tissues presented reduced production of various proinflammatory cytokines, including TNFα, in *GFAP/cFLIP* and *GFAP/cFLIP*_*L*_ relative to controls at 12 weeks of ocular hypertension with no detectable alteration in TUNEL. Besides presenting a similar trend of the proinflammatory versus anti-inflammatory molecules displayed by immunoassays, NanoString-based molecular profiling detected downregulated NF-κB/*RelA* and upregulated *RelB* expression of astroglia in ocular hypertensive samples of *GFAP/cFLIP* compared to ocular hypertensive controls. Analysis of protein expression also revealed decreased phospho-RelA and increased phospho-RelB in parallel with an increase in caspase-8 cleavage products.

**Conclusions:**

A prominent response limiting neuroinflammation in ocular hypertensive eyes with *cFLIP*-deletion in astroglia values the role of cFLIP in the molecular regulation of glia-driven neuroinflammation during glaucomatous neurodegeneration. The molecular responses accompanying the lessening of neurodegenerative inflammation also seem to maintain astroglia survival despite increased caspase-8 cleavage with *cFLIP* deletion. A transcriptional autoregulatory response, dampening RelA but boosting RelB for selective expression of NF-κB target genes, might reinforce cell survival in *cFLIP*-deleted astroglia.

**Supplementary Information:**

The online version contains supplementary material available at 10.1186/s12974-024-03141-4.

## Background

Complex neurodegeneration in glaucoma, a leading cause of blindness [1], encompasses a widespread inflammatory response of glial cells accompanied by progressive loss of retinal ganglion cells (RGCs), optic nerve axons, and their synaptic connections. Despite subtype-specific, topographic, and temporal variations, glial inflammatory responses are evident throughout the visual pathway from the retina to the brain. Although glial responses may initially be beneficial and aid tissue clearance and healing, a shift to chronic activation incites deleterious neuroinflammation and promotes feedback loops, powering neuron loss. Besides increased production of proinflammatory neurotoxic molecules, withdrawal of the glial mechanic, trophic, and bioenergetic support to RGCs convoys glial dysfunction at this stage. Glia-driven stimulation of autoreactive T-cells, autoantibodies, and complement attack may also amplify the damaging stimuli during glaucomatous neurodegeneration (reviewed [2]). Therefore, elucidating the molecular players responsible for regulating glial inflammatory responses remains an essential goal of glaucoma research to develop improved treatments.

Recent studies of postmortem human tissues and animal models have highlighted a significant role for NF-κB as a common transcriptional regulator of the multiple inflammation pathways involved in glia-driven neuroinflammation and neurodegenerative outcomes, including TNFR and TLR signaling, and inflammasome [3–6]. Given the significant role of NF-κB in glia-driven inflammatory responses, following glaucoma studies have continued to explore related regulatory mechanisms further. TNFα, a transcriptional target of NF-κB, is a major proinflammatory cytokine upregulated in the glaucomatous glia [4, 7]. Upon death receptor activation by this specific ligand, TNFR1 signaling, including caspase-8 as an initiator caspase, induces RGC apoptosis, oligodendrocyte death, and axon degeneration [8–10]. 

Beyond promoting RGC death, caspase-8 regulates glia-driven neuroinflammation by mediating glial cytokine production downstream of TNFR and TLR signaling. After death receptor or TLR activation, caspase-8 can raise NF-κB-regulated production of proinflammatory cytokines, including TNFα. Caspase-8 can also promote neuroinflammation by regulating inflammasome involved in the proteolytic release of mature cytokines. Besides its scaffold function for assembly of the signaling complex promoting NF-κB-dependent inflammation, caspase-8 can activate various kinases and inflammatory mediators critical for inflammation signaling and inflammasome-driven processes [11–16]. Extensive evidence has pointed to cFLIP (FLICE-like inhibitory protein, also known as caspase-8 FADD-like apoptosis regulator, Cflar) that functions as a molecular switch between caspase-8-mediated inflammation, apoptosis, or necroptosis pathways [17–19]. Recent experimental studies of glaucoma have also suggested that this caspase-8 homolog making a cytosolic complex with caspase-8 after TNFR or TLR binding regulates diverse processes that control the cell type-specific roles of caspase-8 in cell fate regulations [20]. After homo-oligomerization and auto-processing of procaspase-8, its active form proceeds to the execution of apoptosis via proteolytic caspase cascade. While caspase-8 mediates RGC death, in astroglia, the caspase-8/cFLIP interaction prevents apoptotic cleavage of caspase-8 but regulates the proinflammatory outcomes related to its dead effector domain (DED) or enzymatic activity. In light of this information, this study sought to determine whether deletion of *cFLIP* can eliminate caspase-8-mediated inflammatory responses of astroglia in experimental glaucoma. Analysis of neuroinflammatory outcomes in specific mouse lines with conditional deletion of *cFLIP* (or the long isoform of cFLIP, *cFLIP*_*L*_*)* in astroglia valued cFLIP in the molecular regulation of glia-driven neuroinflammation during glaucomatous neurodegeneration. In addition to a prominent immunomodulatory outcome of astroglial *cFLIP* deletion, we detected a transcriptional autoregulatory response, including induction of RelB, reinforcing cell survival.

## Methods

### Mice

This study included mouse lines with astroglia-targeting conditional deletion of *cFLIP*. The first line targeting glial fibrillary acidic protein (GFAP)-expressing astroglia (named *GFAP/cFLIP*) was generated by breeding the *cFLIP*^*flox*^ [21] (B6.129-*Cflar*^*tm1Ywh*^/J, Stock No: 022009; The Jackson Laboratory, Bar Harbor, ME) [21] into *GFAP-cre/ERT2* (B6.Cg-Tg(GFAP-cre/ERT2)505Fmv/J, Stock No. 012849; The Jackson Laboratory [22]), similar to previous studies [6, 20]. An additional line targeted the long isoform of cFLIP (*cFLIP*_*L*_) in astroglia. The *cFLIP*_*L*_^*flox/flox*^ mice were generated by Dr. Chyuan-Sheng Lin managing the Columbia University, Herbert Irving Comprehensive Cancer Center Genetically Modified Mouse Model Shared Resource. The second line (named *GFAP/cFLIP*_*L*_) generated after cross-breeding of *cFLIP*_*L*_^*flox/flox*^ with *GFAP-cre/ERT2* (The Jackson Laboratory) also allowed for the collection of isoform-specific information.

For conditional recombination, tamoxifen (dissolved in corn oil) was given to crossbreds by intraperitoneal injection (5 mg/40 g mouse) once a day for five consecutive days starting a week before the experimental induction of ocular hypertension. The age- and sex-matched controls (wild-type for the floxed allele with/without cre) received similar tamoxifen injections. Additional controls included the littermates given the oil vehicle only.

PCR with primers detecting *cre/ERT2* (5’-GCC AGT CTA GCC CAC TCC TT-3’, and 5’-TCC CTG AAC ATG TCC ATC AG-3’), *cFLIP*^*flox*^ (5’-CAT GAG CAC TGA GGG ACA CA-3’ and 5’-GCG GAG TTT GCT ACA GGA AG-3’), or *cFLIP*_*L*_^*flox/flox*^ (for the 1st loxp site, L83, 5’-tccaaaggttctaatgcctctt-3’, and 5’-gcagggactgttaactccataa-3’; and for the 2nd loxp site, FL146, 5’-catagggagaccctgtcatcta-3’, and 5’-atggcccatcactcacaaata-3’) alleles verified the genotype. In addition, astroglial cre-recombinase immunolabeling and the analysis of cFLIP protein expression confirmed recombination efficiency.

The mice were housed in a 12-hour light/dark cycle and received standard rodent chow and water *ad libitum*. All animal experiments were conducted according to protocols approved by the Columbia University Institutional Animal Care and Use Committee, and all procedures complied with the Association for Research in Vision and Ophthalmology (ARVO) statement’s tenets for using animals in ophthalmic and vision research.

### Generation of *cFLIP* _*L*_^*flox/flox*^ *mice*

To generate the mice specifically lacking *cFLIP*_*L*_, the exon 6 of the *cFLIP* gene was flanked with two loxP sites (1st loxp: L83; 2nd loxp: FL146). Briefly, the Loxp-Neo-Loxp (LNL) cassette was inserted in the intron upstream of the exon 6 of the *cFLIP* gene on a *B*acterial *A*rtificial *C*hromosome (BAC clone ID: RP23-9D6) by BAC recombineering. The neo cassette was removed by cre recombinase to leave behind the 1st loxp site (L83). A Frt-Neo-Frt-Loxp (FNFL) cassette was inserted in the intron downstream of exon 6 of the *cFLIP* gene. A gene targeting vector was constructed by retrieving the 2 kb left homology arm (5’ to L83), the L83-exon6-FNFL cassette, and the 1.8 kb right homology arm (end of FNFL cassette to 3’) into the pUC57 vector carrying a Diphtheria Toxin Alpha (DTA) chain. The linearized pUC57-Cflip-DTA with AscI was electroporated into KV1 (129B6 hybrid) embryonic stem (ES) cells. Several targeted ES cell clones were identified. These targeted ES cells were injected into C57BL/6J blastocysts to generate germline chimeric mice. Male chimeras were bred to homozygous Actin-beta (ACTB)(Flpe/Flpe) females (B6.Cg-Tg(ACTFLPe)9205Dym/J, Stock No. 005703; The Jackson Laboratory) to transmit the floxed *cFLIP* allele. A detailed floxed *cFLIP* sequence is attached in SnapGene format. This sequence file can be viewed by SnapGene viewer (https://www.snapgene.com/snapgene-viewer/).

### Modeling glaucoma in mouse

Mouse ocular hypertension was experimentally induced by microbead injections into the anterior chamber [23], similar to previous studies [6, 20]. Briefly, 4 µl of a mixture of 6 μm and 1 μm polystyrene microbeads, followed by 1 µl of 10 mg/ml viscoelastic sodium hyaluronate, was injected using a Hamilton syringe (Hamilton Company, Reno, NV). To maintain ocular hypertension for a period of up to 12 weeks, injections were repeated at week 4. The fellow eye was similarly injected with an equivalent volume of physiologic saline. In this commonly utilized experimental model, neuroinflammation develops as a consequence of induced ocular hypertension [6, 20]. 

Intraocular pressure was measured before and after injections and then twice weekly using a TonoLab rebound tonometer (TioLat, Helsinki, Finland) in isoflurane-anesthetized mice. Similar to detailed in previous studies [6, 20], despite steady intraocular pressure levels below 12 mmHg in saline-injected fellow eyes, microbead-injected eyes presented ocular hypertension (28.06 ± 4.12 mmHg) through the 12-week experimental period. The mice with an intraocular pressure increase above 40 mmHg were excluded from the study. The intraocular pressure-time integral was calculated for each ocular hypertensive eye as the mean intraocular pressure over time corrected to the normotensive fellow eye to minimize any influence of intraocular pressure variability among animals. Similar to previous studies [6, 20], ocular hypertensive eyes included in this study had an intraocular pressure-time integral between 200 and 400 mm Hg-days, corresponding to up to 50% neuron loss.

Transgenic effects on glial morphological responses were determined after 12 weeks of ocular hypertension by analyzing the quantitative parameters in retinal whole mounts immunolabeled for GFAP and inflammatory molecules or labeled for the TUNEL assay.

The molecular analysis included 36-plexed immunoassays of retina cytokines and chemokines,

NanoString-based profiling of inflammation-related gene expression in freshly isolated samples of astroglia, and immunoblotting of astroglial proteins for cFLIP expression, phospho-RelA/p65 or phospho-RelB expression, and caspase-8 cleavage. In addition, transgenic effects on neurodegeneration were analyzed by RGC counting.

### Tissue immunolabeling

For morphological analyses of astroglia responses, the expression pattern of the astroglial marker, GFAP, was analyzed as previously described [6, 20]. The whole-mounted retinas fixed in 4% paraformaldehyde for 1 h at room temperature were blocked in 1% BSA (Sigma-Aldrich) and 0.3% Triton X-100 (ThermoFisher Scientific; Waltham, MA) for 1 h incubation. Besides a monoclonal antibody to GFAP (1:500; Abcam; catalog number: ab68428 and ab10062), immunolabeling used a monoclonal antibody to TNFα (1:500; Abcam, catalog number: ab109322). Primary antibody incubation at 4^0^C overnight was followed by another incubation using Alexa Fluor dye (488, 555, or 647)-labeled secondary antibodies (1:1000; ThermoFisher Scientific). Slides were then cover slipped with 50 µl of Fluoroshield mounting medium with 4′,6-diamidino-2-phenylindole, dihydrochloride (DAPI; Abcam, catalog number: ab104139), and images were collected using the laser scanning confocal microscope (Red A1, Nikon Ti Eclipse; Nikon). Similar to previous studies, replacing the primary antibody with serum was used as a negative control or an inappropriate secondary antibody for the species specificity. In addition to whole-mounted retinas, 6-µm thick histological sections of the paraffin-embedded optic nerve head tissues were similarly immunolabeled, as previously described [6, 20]. 

Confocal images of the GFAP-labeled retinal whole mounts were analyzed for quantitative parameters including the intensity (mean pixel intensity reflecting the expression level) and percentage coverage of GFAP labeling (the number of GFAP + pixels divided by the total number of pixels, expressed as a percentage, reflecting the size and density of individual cells), as previously described [6, 20] using ImageJ/FIJI (National Institutes of Health, Bethesda, Maryland). To also determine the spatial distribution of GFAP immunolabeling, after setting the scales to image scales and applying automatic thresholding, the region of interest (ROI) was manually traced for the whole mount area. The center point of each image was next defined as the center of the optic disc to draw a circular ROI with a 100-micrometer radius using X and Y coordinates. 25 consecutive circles were then drawn by increasing the radius from 100 micrometers to 2500 micrometers to obtain annular-shaped ROIs for assessing the mean fluorescence intensity at a particular distance from the optic nerve head. Imaging-based quantitative parameters of GFAP immunolabeling were analyzed in a minimum of 14 mice per group, and the analysis of astroglial TNFα immunolabeling utilized an additional 6 replicates per group. All analyses were conducted by a researcher blinded to the experimental groups.

In addition, a Click-iT™ TUNEL imaging assay kit (ThermoFisher; catalog number: C10245) was utilized to detect apoptotic cells in retinal whole mounts, as previously used [20]. 

### Multiplex protein quantitation

Retinal protein lysates were obtained by homogenization in a lysis buffer (50 mM HEPES-KOH pH 8.0, 100 mM KCl, 2 mM EDTA, 0.10% NP-40, 2 mM dithiothreitol, 10% glycerol, supplemented with protease and phosphatase inhibitors), and protein concentration was measured with a colorimetric Bradford protein assay (BioRad, Hercules, CA), as previously described [6, 20]. Cytokine/chemokine multi-analyte profiling was conducted with a ProcartaPlex 36-plex immunoassay using the Luminex xMAP technology (ProcartaPlex TM mouse cytokine/chemokine panel, Thermo Fisher Scientific). Analyses included triplicated wells of experimental samples and controls, and the standards provided by the kit were used to establish a standard curve for streamlined data analysis with the ProcartaPlex Analysis App. Multiplex immunoassays were repeated three times using samples collected from 3 mice per group in each set.

### NanoString assay

For molecular profiling of the inflammation-related gene expression, NanoString platforms (NanoString Technologies, Seattle, WA, USA) were used to count individual mRNA transcripts in isolated astroglia samples. The astroglia isolation used the two-step immunomagnetic cell selection, as previously described [5, 6, 20]. In the first step, a monoclonal CD11b (1:10; Abcam, catalog number: ab8878) was used to select microglia from papain-dissociated retinas. The second step isolated astroglia from the microglia/macrophage-depleted cell suspension using a monoclonal antibody to GLAST1/ACSA-2 (1:10; Miltenyl Biotech, catalog number 130-099-138). RT-PCR and immunoblotting of selected samples for specific markers verified this technique yielding ∼ 95% purity [5, 6, 20, 24]. Similar to previous studies, samples were pooled from multiple eyes matched for cumulative intraocular pressure exposure (and neuron counts within each group) [5, 6, 20]. 

RNA was extracted from freshly isolated samples using a RNeasy Mini Kit (Qiagen, Germantown, MD), and Nanostring reactions were prepared according to the manufacturer’s recommendation for the nCounter Mouse Inflammation V2 Panel (NanoString Technologies). The panel consisted of 248 inflammation-related genes and 6 internal reference genes (Cltc, Gapdh, Gusb, Hprt, Pgk1, Tubb5), 8 negative and 6 positive controls for normalization and the control of technical variability. The RNA integrity was assessed in all samples by nucleic acid fragmentation using a bioanalyzer (Agilent, Santa Clara, CA), and the RNA quantity was assessed using a Qubit fluorometer. Two hundred ng total RNA (RNA integrity > 8.3; minimum concentration, 66–83 ng/µL) per sample was submitted for hybridization and immobilizing cartridge processing. The gene expression data collected using the nCounter Digital Analyzer (NanoString Technologies) were analyzed using the nSolver Analysis Software 3.0 (NanoString Technologies). Heatmaps and volcano plots were generated for group comparisons in gene expression profiling using the fold change in normalized molecular counts and z-scores. NanoString assay used three samples collected from approximately 5 mice per group in each set.

### Immunoblotting

Immunoblotting followed similar protocols to previous studies [6, 20]. cFLIP expression was analyzed using a monoclonal antibody (1:500; ThermoFisher Scientific, catalog number: MA5-15739) To analyze caspase-8 cleavage, an antibody to procaspase-8 and cleaved subunits (1:500; ThermoFisher Scientific, catalog number: 13423-1-AP) was used. The primary antibodies also included phosphorylation site-specific antibodies to p65 [phospho-Ser536] (1:500; Abcam, catalog number: ab76302) or RelB [phospho-Ser552] (1:500; ThermoFisher Scientific, catalog number: PA5-36837). In addition, a β-actin antibody (1:1000; ThermoFisher Scientific, catalog number: MA5-15739, and Abcam, catalog number: ab179467) was used for loading and transferring controls. After incubation with infrared dye-labeled secondary antibodies (1:10,000; Li-Cor, Lincoln, NE) for 1 h, proteins were visualized using Odyssey Infrared Imaging system (Li-Cor), and the band intensity values were normalized to β-actin. Immunoblotting was repeated at least three times with samples collected from approximately 40 mice per group.

### RGC counting

RGCs were counted in retinal whole mounts immunolabeled for RNA-binding protein with multiple splicing (RBPMS, 1:200; GeneTex, Irvine, CA; catalog number: GTX118619), as previously described [6, 20]. Laser scanning confocal microscopy (Red A1, Nikon Ti Eclipse; Nikon, Melville, NY) and the NIS-elements AR5 software (Nikon) were used to acquire non-overlapping tile images of the whole-mounted retina for a depth of 0–30 μm as z stacks (with 5 μm step size) at a magnification of 10, and images were collapsed into two-dimensional images with maximum intensity projections for RGC counting. RGC counting was conducted in a minimum of 16 mice per group. All images were masked for the experimental group of samples. Besides RBPMS labeling, RGC counting criterion included a minimal soma size of 10 μm to eliminate dying or phagocytized RGCs, as previously described [6, 20]. 

### Statistical analysis

Statistical analysis of the experimental data was carried out in a masked fashion using SigmaPlot software (version 12.5; Systat Software, Inc., San Jose, CA). Group differences were compared using the one-way analysis of variance (ANOVA) followed by the pairwise multiple comparison procedure of the Holm-Sidak method or the Kruskal-Wallis one-way ANOVA on ranks followed by the Tukey test. The data are presented as mean ± SD along with the p-values. A p-value of less than 0.05 was considered significant.

## Results

### Verification of mouse lines

**Fig. 1:**
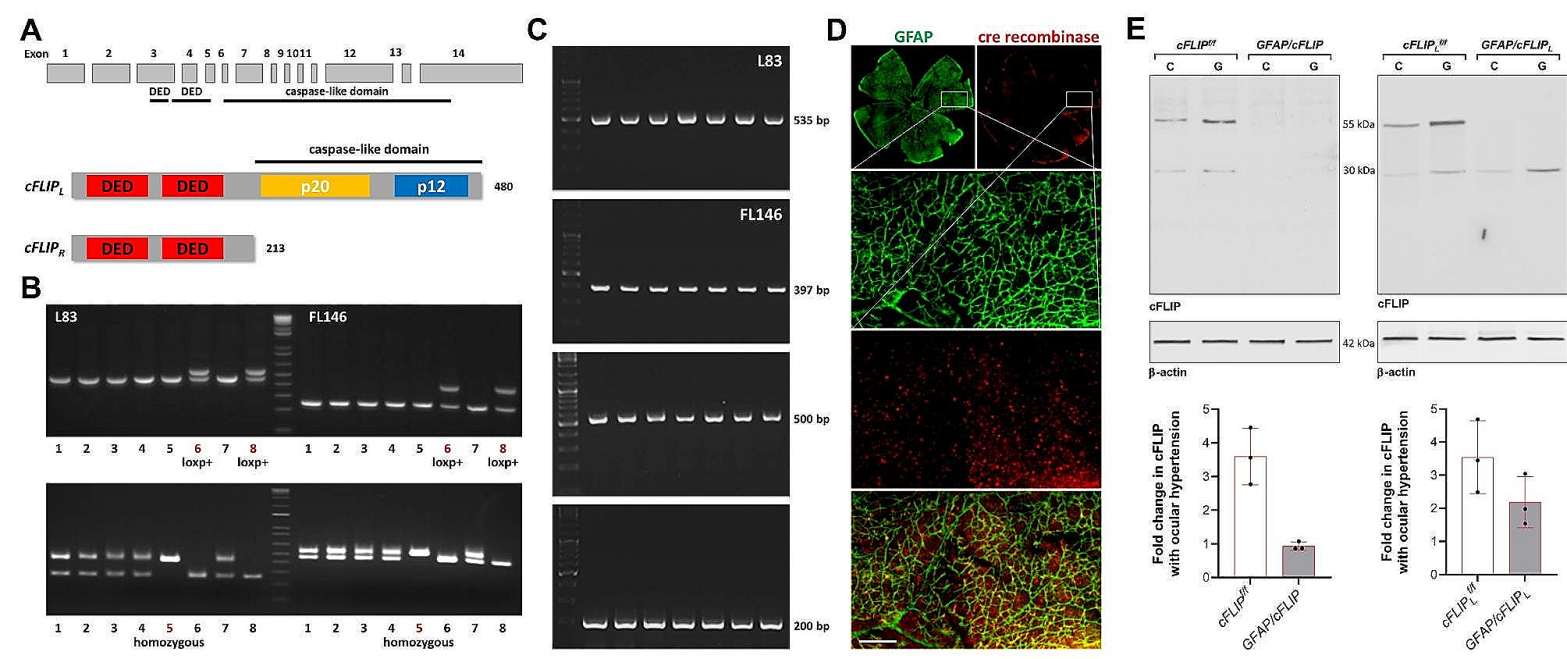
Generation of *GFAP/cFLIP*_*L*_ mice. **(A)** Diagram of the *cFLIP* gene. **(B)** PCR screening. After breeding of the male chimeras and the ACTB(Flpe/Flpe) females, screening of 8 pups for L83 and FL146 by agarose gel electrophoresis of PCR products showed loxp heterozygous male (#6 and #8). After pairing the heterozygous males with C57BL/6J females, screening of 8 pups from crossbreeding of the heterozygous mice showed loxp homozygous (#5). **(C)** Continued genotyping verified L83 and FL146 in *cFLIP*_*L*_ mutants. Additional PCRs show *GFAP-Cre/ERT2* (500 bp) and mutant *cFLIP* (200 bp). **(D)** Cre recombinase expression (red) was also analyzed in retinal GFAP-expressing astroglia (green) by specific immunolabeling of retinal whole mounts. White boxed areas are shown in higher magnification, and the bottom panel shows the merged images of GFAP (green) and cre recombinase (red) immunolabeling (scale bar, 100 μm). **(E)** Immunoblots of retinal astroglia proteins detected no band corresponding to long (∼ 60 kDa) or short (∼ 30 kDa) isoforms of cFLIP in *GFAP/cFLIP*. However, the short isoform was detectable in *GFAP/cFLIP*_*L*_. Also note increased band intensities in experimental glaucoma **(G)** samples compared to non-glaucomatous controls (C). Graphs present data (mean ± SD) from quantitative analysis of the band intensities normalized to β-actin

This study tested whether conditional deletion of *cFLIP* in astroglia can reduce neuroinflammation in experimental glaucoma. Mouse *cFLIP* codes two isoforms, cFLIP_L_ and cFLIP_R_ [25, 26]. Figure 1 shows a diagram of the *cFLIP* gene in panel A. Besides targeted *cFLIP* deletion, in which exon 1 (used by both c-FLIP_L_ and c-FLIP_R_) was flanked [21], a new line was generated for the selective deletion of *cFLIP*_*L*_ by flanking the exon 6 of the *cFLIP* gene with two loxP sites to determine isoform-specific differences. Panels B and C of Fig. 1 present the PCR screening data, verifying the two loxP sites, L83 and FL146, by agarose gel electrophoresis of PCR products in *cFLIP*_*L*_ mutants. PCR genotyping also verified GFAP-Cre/ERT2 (500 bp) and mutant cFLIP (200 bp). In addition, astroglia-targeting cre/lox-based deletion efficiency was tested by cre recombinase immunolabeling in GFAP-expressing astroglia in retinal whole mounts (Fig. 1D). Figure 1 also presents immunoblots of the isolated astroglia samples verifying the change in cFLIP protein expression. Although astroglia from controls exhibited cFLIP expression, including both cFLIP_L_ and cFLIP_R_ isoforms, no prominent cFLIP or cFLIP_L_ immunoreactivity was detectable in transgenic samples. As presented in panel E of Fig. 1, immunoblots of retinal astroglia proteins detected no band corresponding to long (∼ 60 kDa) or short (∼ 30 kDa) isoforms of cFLIP in *GFAP/cFLIP*. However, retinal astroglia proteins isolated from *GFAP/cFLIP*_*L*_ presented ∼ 30 kDa immunoreactivity corresponding to cFLIP_R_. These data collectively support the effectiveness of targeted deletions in astroglia.

### Transgenic effects on cellular apoptosis in *cFLIP*-deleted astroglia

**Fig. 2:**
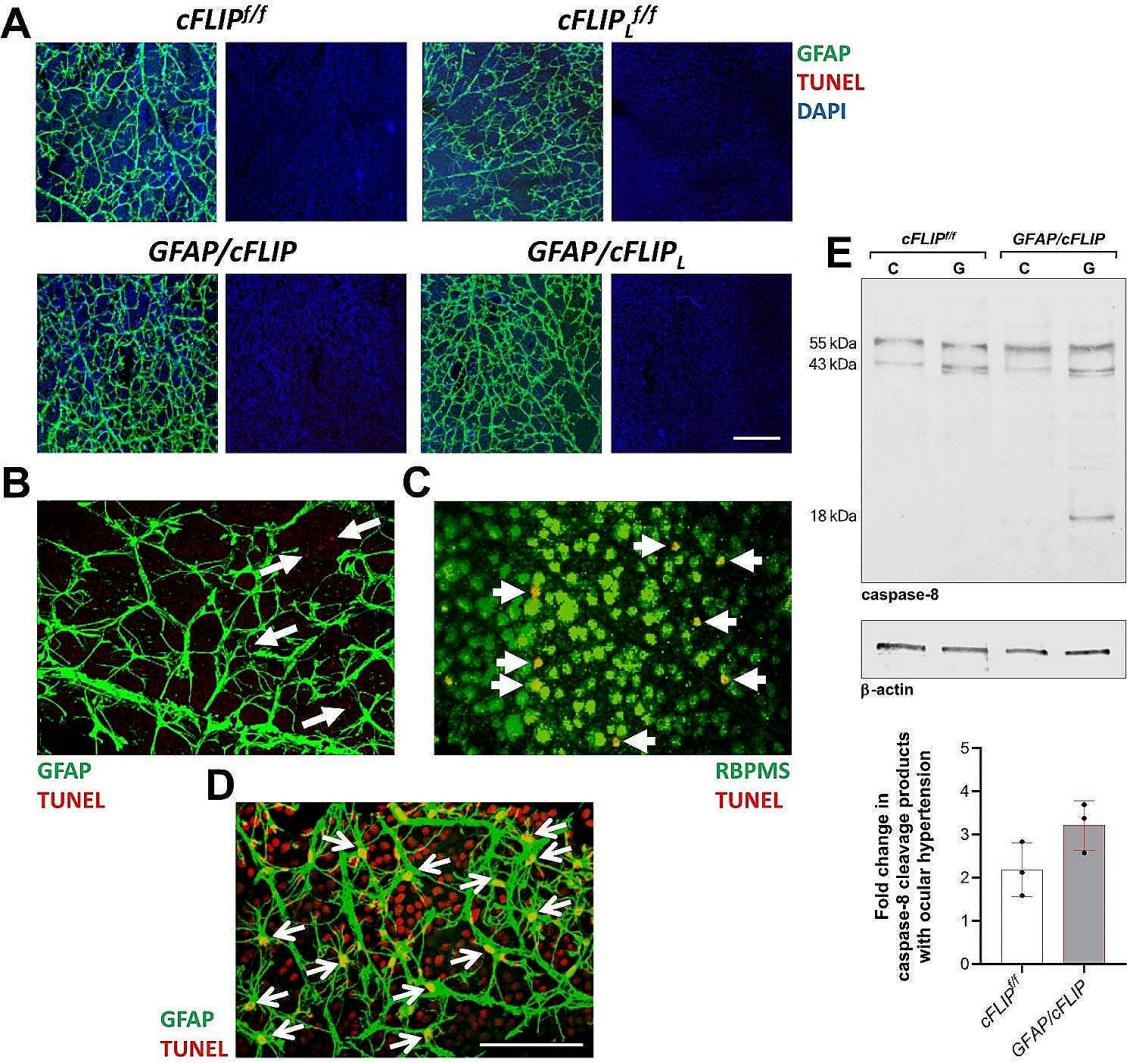
Analysis of astroglial apoptosis. **A.** No alteration was detectable in TUNEL (red) of GFAP + astroglia (green) in retinal whole mounts from ocular hypertensive *GFAP/cFLIP* or *GFAP/cFLIP*_*L*_ compared to ocular hypertensive controls, *cFLIP*^*f/f*^ or *cFLIP*_*L*_^*f/f*^ (scale bar, 100 μm). When repeated in a minimum of 6 samples per group, TUNEL did not detect astroglia apoptosis. Blue shows DAPI nuclear stain. **B.** Some TUNEL + cells, not associated with GFAP-labeled astroglia, were detectable in some regions of the underlying layer of retinal ganglion cells (white arrows). **C.** Although GFAP-labeled astroglia were negative for TUNEL, additional analysis demonstrated TUNEL in some RBPMS (green)-labeled retinal ganglion cells (white short arrows). **D**. As a positive control, DNase I treatment of the whole-mounted retina resulted in TUNEL of retinal cells, including GFAP + astroglia (white thin arrows). **E.** Despite no alteration in astroglia TUNEL with *cFLIP* deletion, Western blot analysis detected an increase in caspase-8 cleavage products in experimental glaucoma (G) samples compared to non-glaucomatous controls (C). Besides the full-length 55 kDa inactive proenzyme, immunoblot analysis of caspase-8 expression detected p43 and p18 subunits in astroglia samples isolated from ocular hypertensive *GFAP/cFLIP*. Immunoblotting was repeated at least three times with samples collected from approximately 40 mice per group. The graph presents the fold change data (mean ± SD) from quantitative analysis of the band intensities normalized to β-actin

Since cFLIP regulates caspase-8-mediated cell death and inflammation pathways, Western blotting first focused on apoptotic caspase-8 activity in the *cFLIP*-deleted astroglia. Western blot analysis testing caspase-8 expression in the isolated astroglia proteins detected some cleavage products in ocular hypertensive samples besides the full-length 55 kDa inactive proenzyme. In addition to p55 procaspase-8 and its p43 cleavage product in control samples, immunoblots detected p18 in *cFLIP*-deleted ocular hypertensive samples. Figure 2 presents these immunoblots and the related quantitative data (panel E). Despite increased caspase-8 cleavage, no transgenic effect was detectable on cellular apoptosis in astroglia. As shown in Fig. 2A, there was no change in TUNEL of astroglia between ocular hypertensive *GFAP/cFLIP* or *GFAP/cFLIP*_*L*_ and ocular hypertensive controls. Some TUNEL + cells were detectable in the underlying layer of RGCs; however, this labeling was not associated with GFAP-labeled astroglia (Fig. 2B). Instead, additional analysis demonstrated colocalization of TUNEL with RBPMS, a cell marker for RGCs (Fig. 2C) known to undergo apoptosis in ocular hypertensive eyes. As a positive control, DNase I treatment of the whole-mounted retina resulted in TUNEL of retinal cells, including GFAP + astroglia (Fig. 2D). In support of the maintained survival of astroglia after targeted deletion of *cFLIP* or *cFLIP*_*L*_, further analysis in *GFAP/cFLIP* or *GFAP/cFLIP*_*L*_ mice detailed below also demonstrated protected cell structure and morphological features accompanied by continued gene and protein expression in these cells.

### Transgenic effects of the ocular hypertension-induced inflammatory responses of astroglia with *cFLIP*, or *cFLIP*_*L*_, deletion

**Fig. 3:**
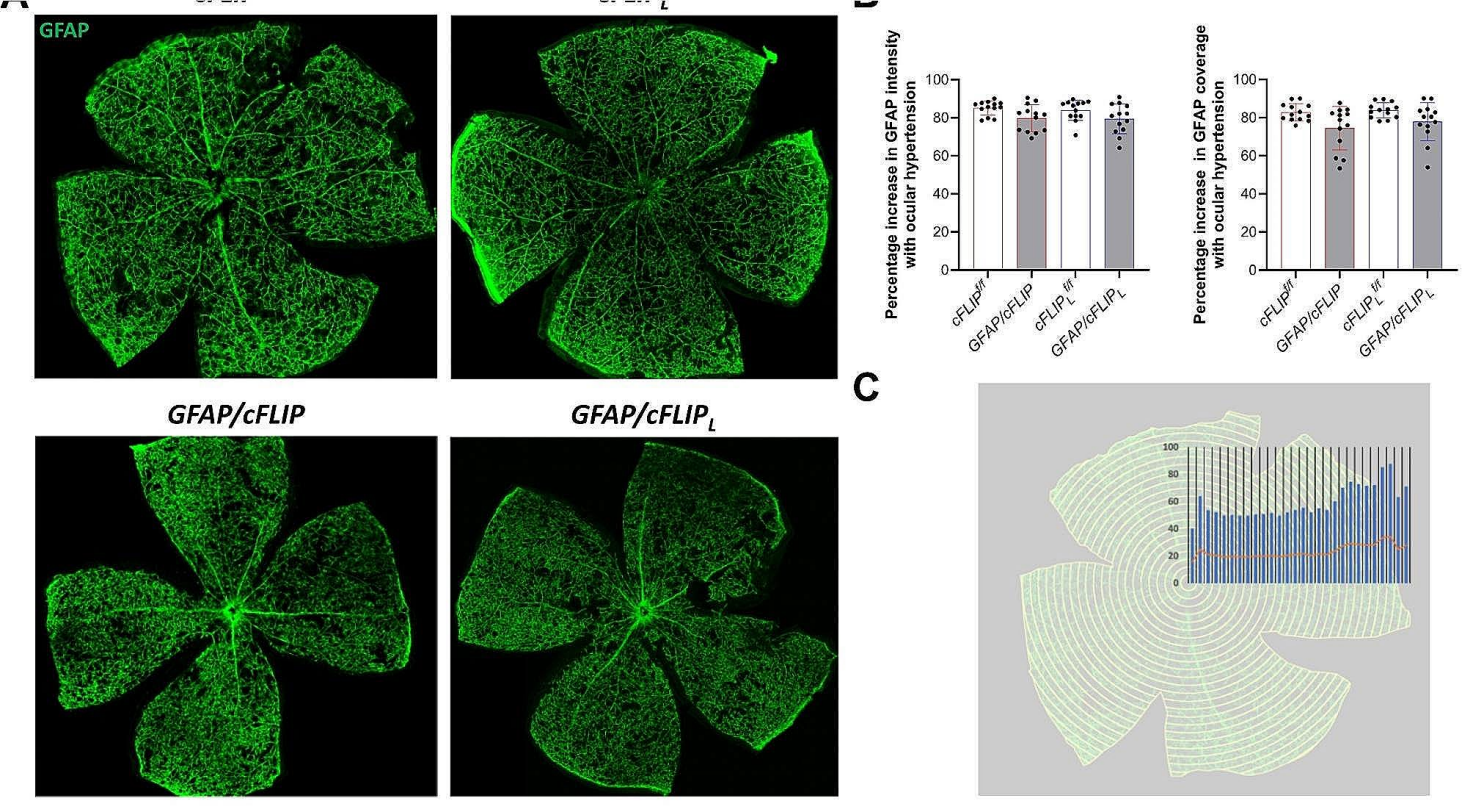
Morphological analysis of the astroglia response. **(A)** Retinal whole mounts immunolabeled for GFAP showed subtle alterations in the quantitative morphological parameters of astroglial reactivity in ocular hypertensive *GFAP/cFLIP* and *GFAP/cFLIP*_*L*_ compared to ocular hypertensive controls (*cFLIP*^*f/f*^ and *cFLIP*_*L*_^*f/f*^). **(B)** Graphs show the intensity and the percentage coverage of GFAP immunolabeling in different groups. The data (mean ± SD) represent a minimum of 14 mice per group. Both GFAP intensity and coverage were not significantly different in ocular hypertensive *GFAP/cFLIP* or *GFAP/cFLIP*_*L*_ than in ocular hypertensive controls (one-way ANOVA; *p* > 0.05). **(C)** The studied quantitative parameters of GFAP immunolabeling did not also present significant spatial alterations (one-way ANOVA; *p* > 0.05)

Next, to determine transgenic effects on astroglia-driven neuroinflammation, alterations in morphological and molecular responses of astroglia were analyzed in *GFAP/cFLIP*, *GFAP/cFLIP*_*L*_, and control mice with or without experimentally induced glaucoma. When the GFAP immunolabeling pattern of astroglia was studied in whole-mounted retinas, reactive astroglia in ocular hypertensive samples exhibited increased intensity and percentage coverage of GFAP labeling as expected. Compared to ocular hypertensive *cFLIP*^*f/f*^ or *cFLIP*_*L*_^*f/f*^, ocular hypertensive *GFAP/cFLIP* or *GFAP/cFLIP*_*L*_ presented faint alterations in these quantitative parameters. The intensity of GFAP immunolabeling, reflecting the level of GFAP expression, presented only a subtle drop in ocular hypertensive *GFAP/cFLIP* and *GFAP/cFLIP*_*L*_ relative to ocular hypertensive controls. Similarly, ocular hypertension-induced increase in the percentage GFAP coverage, reflecting the size and density of individual cells, slightly decreased with astroglial *cFLIP or cFLIP*_*L*_ deletion. However, neither these morphological data nor the spatial distribution of the GFAP immunolabeling detected no significant difference between transgenic and control groups (*p* > 0.05). Figure 3 exemplifies GFAP immmunolabeling in control and transgenic groups (panel A) and presents the quantitative image analysis data (panels B and C).

**Fig. 4:**
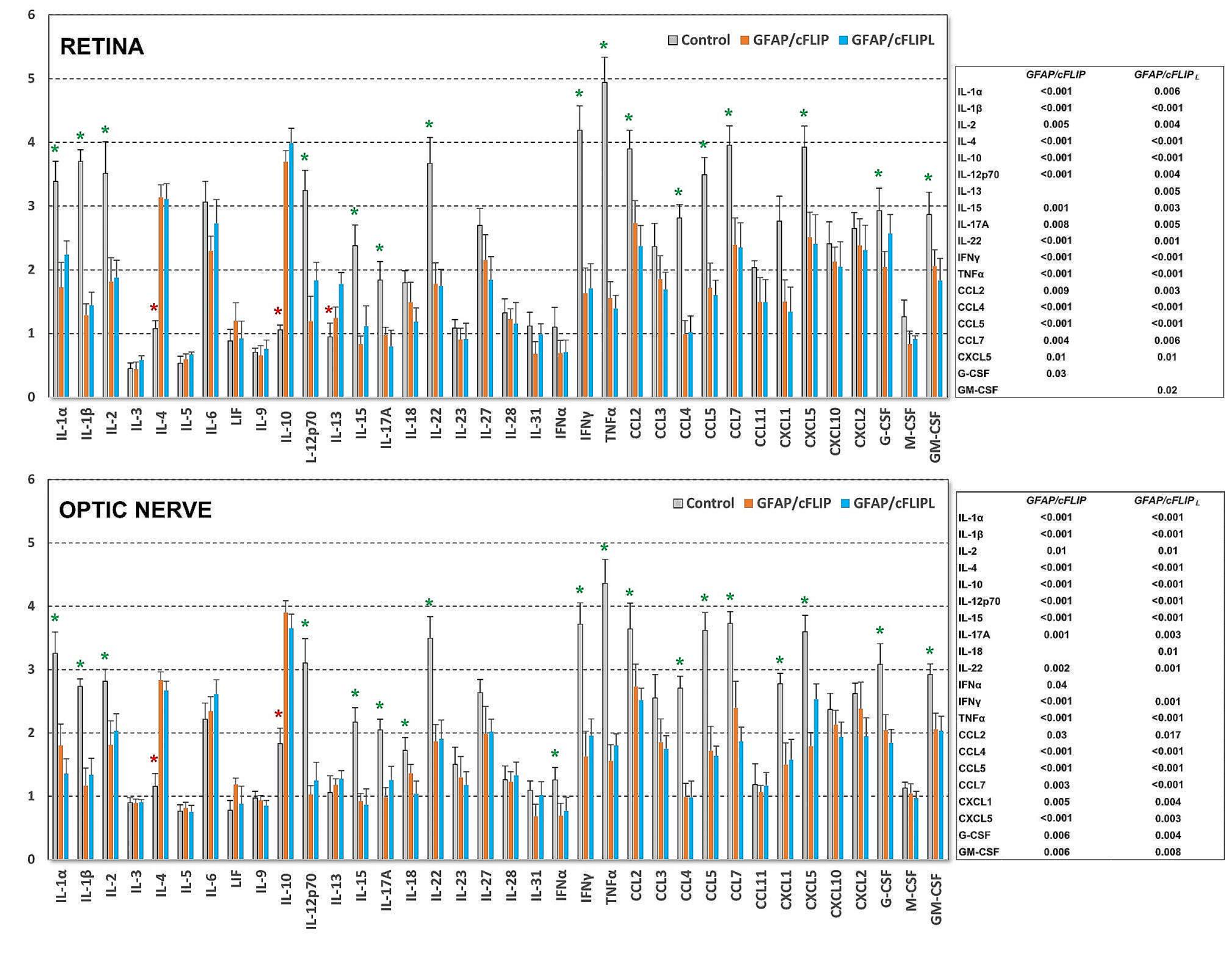
Cytokine/chemokine profiling. Multiplex immunoassays of retina and optic nerve proteins detected altered titers of multiple cytokines and chemokines in ocular hypertensive *GFAP/cFLIP* and *GFAP/cFLIP*_*L*_ mice than ocular hypertensive controls. Graphs present the fold change in ocular hypertension-induced alterations in 36 molecules studied in *GFAP/cFLIP*, *GFAP/cFLIP*_*L*_, and controls. The data (mean ± SD) represent a minimum of 3 samples collected from 3 mice per group in each set. Green and red asterisks show significantly decreased or increased molecules, respectively, and accompanying tables list these molecules along with the *p*-values of group comparisons using one-way ANOVA

The study also included cytokine and chemokine profiles in the retina and optic nerve samples using multiplex immunoassays. Luminex-based analysis showed decreased titers of multiple cytokines and chemokines in ocular hypertensive *GFAP/cFLIP* and *GFAP/cFLIP*_*L*_ mice compared to ocular hypertensive controls at 12 weeks of ocular hypertension. The proinflammatory cytokines, including IL1, IL2, IFNγ, and TNFα, presented significantly (*p* < 0.001) lower titers, while the titers of the anti-inflammatory cytokines, including IL4 and IL10, were significantly (*p* < 0.001) increased in *GFAP/cFLIP* and *GFAP/cFLIP*_*L*_. Figure 4 shows the related data.

**Fig. 5:**
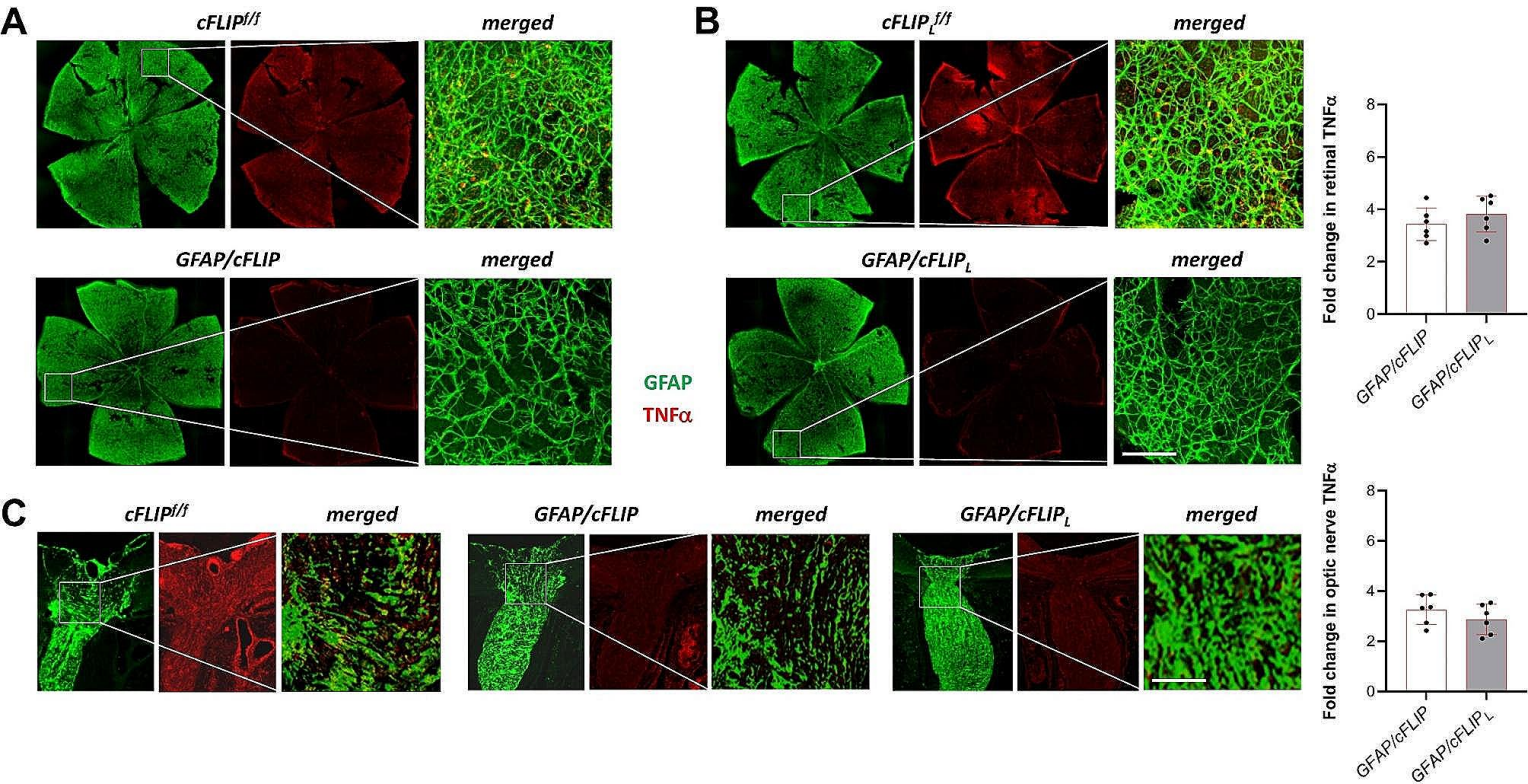
Tissue immunolabeling of TNFα. **(A)** GFAP (green) and TNFα (red) immunolabeling of retinal whole mounts demonstrated a prominent decrease in ocular hypertensive *GFAP/cFLIP* compared to ocular hypertensive controls ocular hypertensive controls (*cFLIP*^*f/f*^). **(B)** GFAP (green) and TNFα (red) immunolabeling of retinal whole mounts was similarly decreased in ocular hypertensive *GFAP/cFLIP*_*L*_ compared to ocular hypertensive controls. **(C)** Optic nerve sections similarly exhibited decreased TNFα immunolabeling in ocular hypertensive *GFAP/cFLIP*. The merged images of the white boxed areas are shown in higher magnification. Images are representative of a minimum of 6 samples per group (scale bar, 100 μm). Graphs present data (mean ± SD) from quantitative analysis of TNFα immunolabeling in GFAP + astroglia in transgenic and control samples. The intensity of astroglial TNFα immunolabeling was significantly lower in ocular hypertensive *GFAP/cFLIP* or *GFAP/cFLIP*_*L*_ than in ocular hypertensive controls (one-way ANOVA; *p* < 0.001)

To support the immunoassay data, the following analyses included tissue immunolabeling. TNFα immunolabeling was analyzed in retinal whole mounts and optic nerve sections to assess the proinflammatory phenotype of astroglia. As shown in Fig. 5, a prominent decrease was detectable in the TNFα labeling of GFAP + astroglia in ocular hypertensive *GFAP/cFLIP* and *GFAP/cFLIP*_*L*_ mice relative to ocular hypertensive controls. Quantitative analysis indicated an approximately three-fold decrease in TNFα immunolabeling of the retina (Fig. 5A and B) and optic nerve (Fig. 5C) in *GFAP/cFLIP* and *GFAP/cFLIP*_*L*_. Figure 5 also presents the quantitative data from image analysis of TNFα immunolabeling in the retina and optic nerve tissues, indicating an up to four-fold decrease with *cFLIP* or *cFLIP*_*L*_ deletion in astroglia (*p* < 0.001). Thus, deletion of *cFLIP*, or *cFLIP*_*L*_, limited proinflammatory responses of astroglia.

### Additional molecular profiling in *cFLIP*-deleted astroglia

**Fig. 6:**
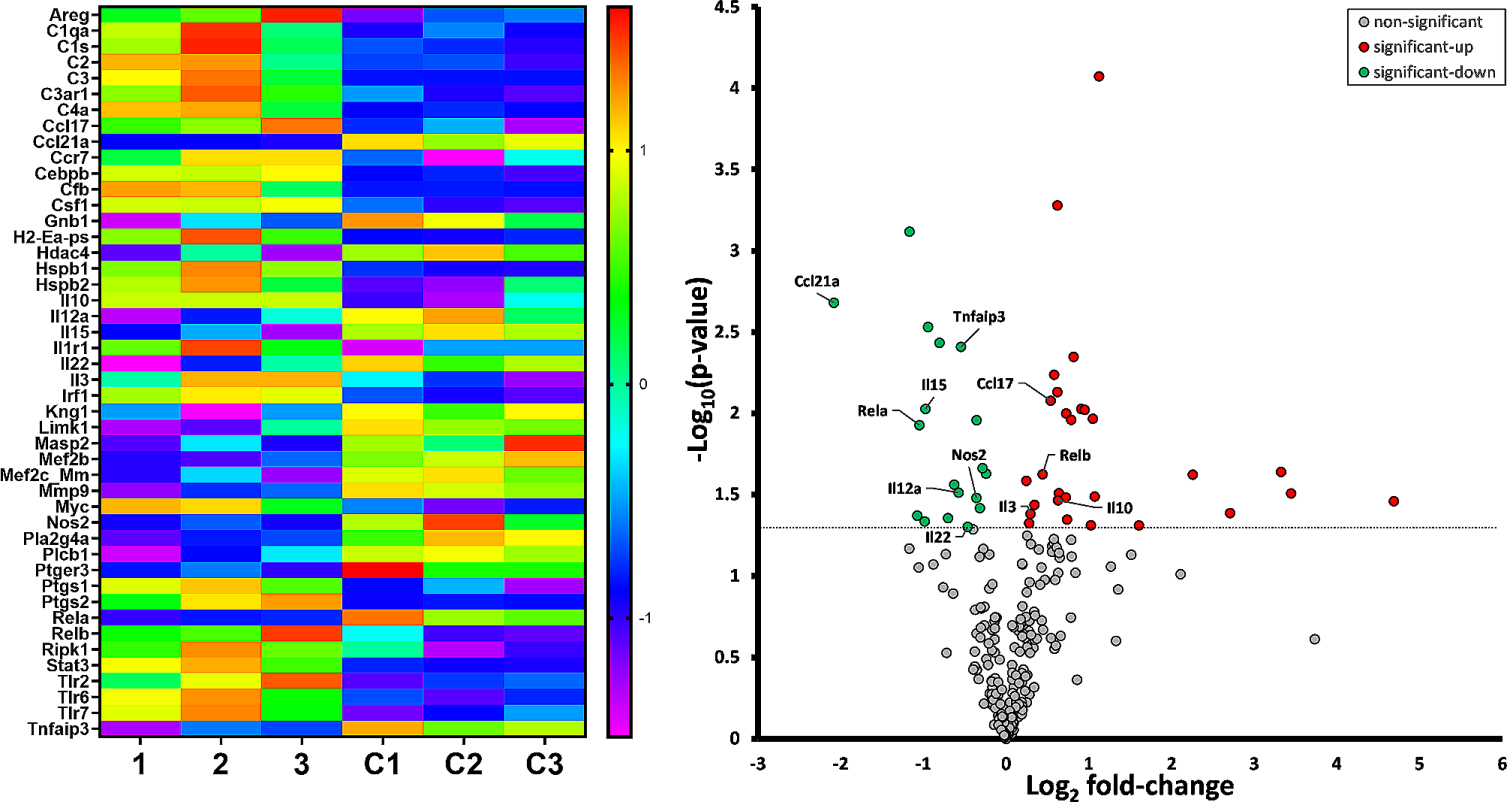
NanoString-based molecular analysis of the astroglia response. NanoString profiling of 248 inflammation-related genes in isolated astroglia samples detected multiple genes significantly upregulated or downregulated in ocular hypertensive *GFAP/cFLIP* mice (1–3) compared to ocular hypertensive controls (C1-3). The heatmap reflects z-scores for molecules with significantly changed expression. The volcano plot presents the fold change data. The dotted line shows significance set at *p* < 0.05; the red data points above the reference line represent increased, and the green data points represent decreased expression. NanoString assay used a minimum of three samples collected from approximately 5 mice per group in each set

NanoString**-**based profiling of the inflammation-related genes explored the molecular responses of astroglia further. Transcripts from multiple genes were significantly upregulated or downregulated in astroglia samples isolated from *GFAP/cFLIP* mice compared to controls. Heatmaps and volcano plots of the molecular data are presented in Fig. 6. Compared to ocular hypertensive *cFLIP*^*f/f*^ controls, the retinal astroglia isolated from ocular hypertensive *GFAP/cFLIP* presented a significant increase or decrease in 46 out of 248 genes studied. The molecular alterations exhibited a trend parallel to that detected by immunoassays and tissue immunolabeling. Significantly increased molecules in *cFLIP*-deleted astroglia included an anti-inflammatory cytokine, *Il10* (*p* = 0.01), while several proinflammatory cytokines, including *Il12* (*p* = 0.03), *Il15* (*p* = 0.01), and *Il22* (*p* = 0.04) were decreased. Other molecules decreased in *cFLIP*-deleted astroglia included a regulator molecule of TNFα signaling, *Tnfaip3* (*p* = 0.003), and *nos2* (*p* = 0.01). Several complement components were among the molecules with increased expression in *cFLIP*-deleted astroglia.

**Fig. 7:**
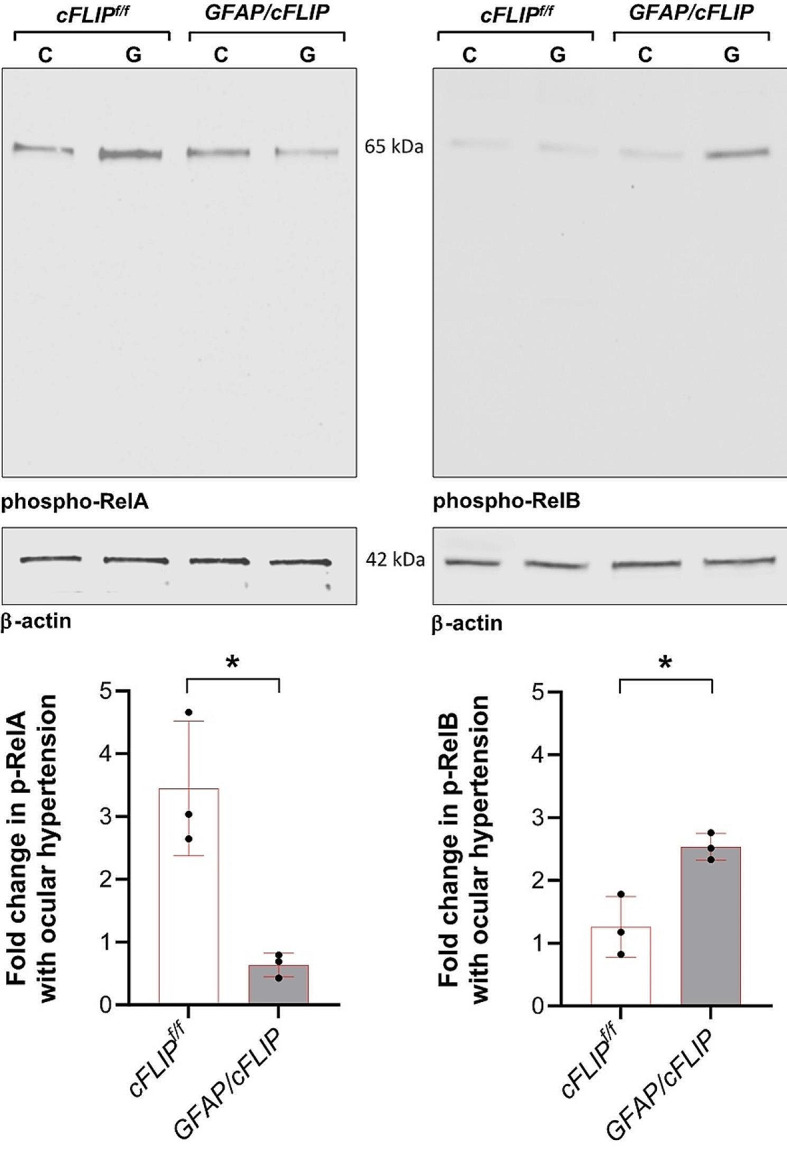
Western blot analysis of molecular responses. Western blots of astroglia proteins used phosphorylation site-specific antibodies to analyze phospho-RelA (p65) and phospho-RelB expression in transgenic and control mice with experimental glaucoma (G) compared to non-glaucomatous controls (C). This analysis detected decreased phospho-RelA and increased RelB in ocular hypertensive *GFAP/cFLIP* compared to ocular hypertensive controls (*cFLIP*^*f/f*^). Immunoblotting was repeated at least three times with samples collected from approximately 40 mice per group. Graphs present the fold change data (mean ± SD) from quantitative analysis of the β-actin-normalized band intensities in transgenic and control samples (*one-way ANOVA; *p* = 0.01)

An exciting outcome of the NanoString-based gene profiling in ocular hypertensive samples included NF-κB components, as *RelA* (p65) decreased (*p* = 0.002) and *RelB* (*p* = 0.02) showed increased RNA counts in *GFAP/cFLIP* mice compared to controls. To further evaluate these molecular responses, Western blot analysis evaluated protein expression in isolated astroglia samples from *GFAP/cFLIP*, *GFAP/cFLIP*_*L*,_ and controls. When phosphorylation site-specific antibodies were used to analyze phospho-RelA (p65) and phospho-RelB expression, retinal astroglia exhibited decreased expression of phospho-RelA and increased expression of RelB in ocular hypertensive samples of *GFAP/cFLIP* compared to ocular hypertensive controls. Figure 7 exemplifies related immunoblots and presents the statistical comparison of the protein expression in transgenic and control samples. Besides alterations in proinflammatory versus anti-inflammatory molecules, these findings support a transcriptional autoregulatory response, reducing RelA but enhancing RelB in *cFLIP*-deleted astroglia.

### Transgenic effects on neuron survival in experimental glaucoma

**Fig. 8:**
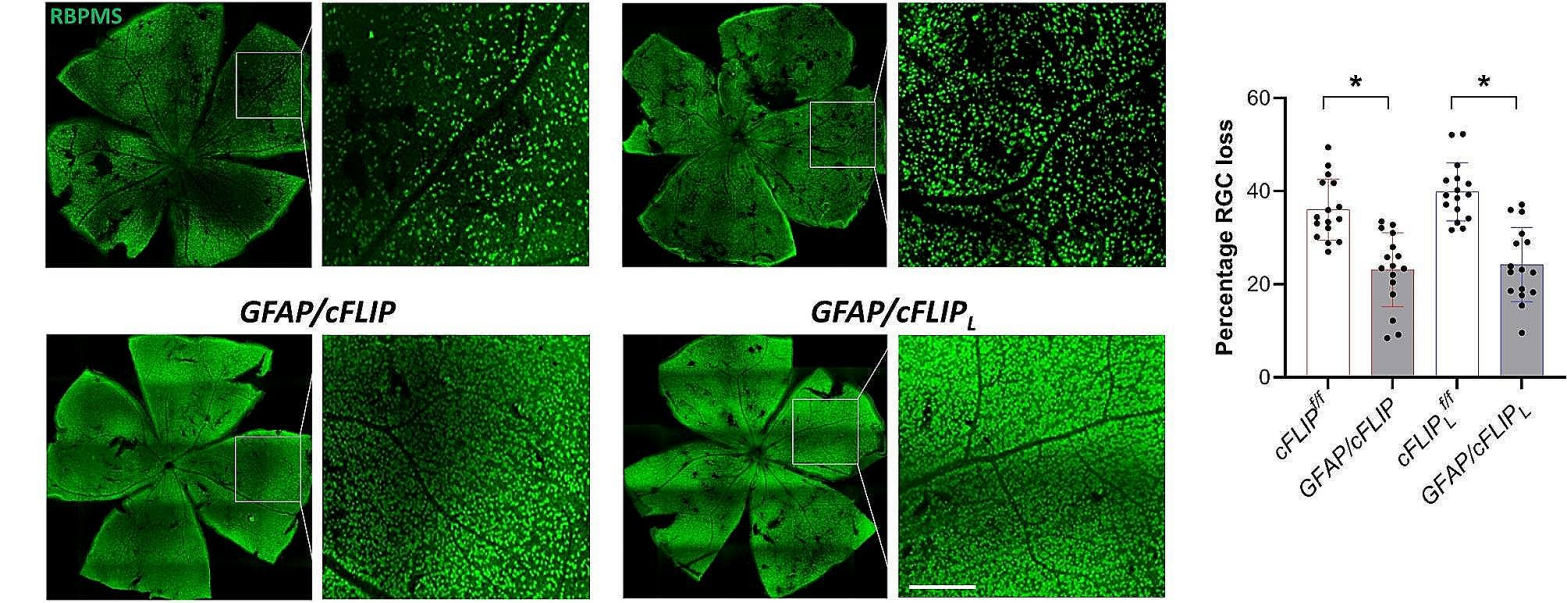
RGC counts. **(A)** Counting of the RBPMS-labeled RGCs (green) in retinal whole mounts detected a greater number of remaining RGCs in ocular hypertensive *GFAP/cFLIP* and *GFAP/cFLIP*_*L*_ compared to ocular hypertensive controls (*cFLIP*^*f/f*^ and *cFLIP*_*L*_^*f/f*^), corresponding to approximately 36 and 39% protection against RGC loss in experimental glaucoma. White boxed areas are shown in higher magnification (scale bar, 200 μm). **(B)** Graphs show the percentage loss in RGC counts in different groups (*one-way ANOVA; *p* < 0.001). The data (mean ± SD) represent a minimum of 16 mice per group

To determine transgenic effects on the status of ocular hypertension-induced neurodegeneration, RBPMS-labeled RGCs were counted in whole-mounted retinas. Figure 8 exemplifies RBPMS immunolabeling of retinal whole mounts in control and transgenic groups (panel A) and presents the comparative RGC loss data (panel B). RGC counting detected a greater number of remaining RGCs in ocular hypertensive *GFAP/cFLIP* (42,231 ± 4,079; ∼23% neuron loss versus 34,562 ± 3,503; ∼36% neuron loss; *p* < 0.001) and *GFAP/cFLIP*_*L*_ (41,229 ± 3,986; ∼24% neuron loss versus 32,671 ± 3,543; ∼39% neuron loss; *p* < 0.001) mice than ocular hypertensive controls. As opposed to the RGC loss in controls, these values correspond to approximately 36% and 39% protection in *GFAP/cFLIP* and *GFAP/cFLIP*_*L*_, respectively.

## Discussion

Although therapeutic immunomodulation is a logical strategy to avoid inflammatory neurotoxicity in several neurodegenerative diseases, including glaucoma, molecular regulation of neuroinflammation has yet to be fully elucidated to identify effective treatment targets. Based on recent research findings from the studies of animal models and the postmortem tissues of glaucomatous human donors, upregulated molecules in the glaucomatous glia include several inflammatory mediators linked to TNFR signaling, TLR signaling, and inflammasome activation [2, 27]. Among the glia-produced proinflammatory cytokines that function as effectors of neuroinflammation, TNFα comes forward as a prominent contributor to the proinflammatory-to-anti-inflammatory cytokine imbalance in human glaucoma and experimental models [4, 7]. It has also become evident that besides the direct neurotoxicity of TNFα to RGCs through dead receptor signaling, downstream NF-κB activation and transcription of immune mediators amplify the vicious cycle of neurodegenerative inflammation driven by glia. A more recent study of experimental glaucoma in mice with astroglia-targeting cre/lox-based conditional inhibition of NF-κB activation supported a critical role for this transcriptional activator in neuroinflammatory outcomes [6]. The following glaucoma studies have also built a scientific basis for a regulatory role of cFLIP, a gene target of NF-κB, in glia-driven neuroinflammation. Upon activation of immune receptors, the interaction of DED-containing proteins, caspase-8 and cFLIP, appears to constitute a signaling checkpoint for cell fate decisions. [20] The current study focused on investigating the importance of cFLIP for astroglia-driven neuroinflammation in experimental glaucoma using conditional *cFLIP* knockouts. Besides *cFLIP*, this study analyzed the outcomes of the selective deletion of *cFLIP*_*L*_ in astroglia since the balance between different isoforms, cFLIP_L_ and cFLIP_R_, appears to impact the regulatory activities [28, 29]. The findings of this study demonstrated a decreased inflammatory response in experimental glaucoma with *cFLIP, or cFLIP*_*L*_, deletion in astroglia. This immunomodulatory outcome was accompanied by regulatory molecular responses maintaining astroglia survival. Lessening of neuroinflammation by targeted deletion of astroglial *cFLIP, or cFLIP*_*L*_, provided RGC protection in ocular hypertensive eyes, supporting the potential for immunomodulation in glaucoma treatment.

### Caspase-8/cFLIP interaction in astroglia

This study, stimulated by recent experimental findings implying caspase-8/cFLIP interaction in cell fate decisions in experimental glaucoma [20], assessed an essential role of cFLIP in the molecular regulation of glia-driven neuroinflammation. In the extrinsic pathway of RGC apoptosis in glaucoma, which can be triggered by the ligation of dead receptors, such as TNFR1 [7] or Fas [30], caspase-8 plays an initiator role. After the engagement of dead receptors, the initial membrane-bound complex rapidly signals for receptor-interacting protein kinase 1 (RIPK1)-mediated activation of NF-κB that promotes cell survival and proinflammatory signaling. In contrast, a cytoplasmic complex formed in a second step promotes apoptosis. Recruitment of procaspase-8 to the death-inducing signaling complex leads to homo-oligomerization and initiates an autocatalytic multistep cleavage process, producing the mature enzyme. The autoactivated caspase-8 cleaves effector caspases in the downstream apoptosis cascade [31, 32]. However, procaspase-8 zymogen and its partially cleaved form can also exert pro-survival and proinflammatory activities through RIPK1, while preventing the necroptotic RIPK activity [11, 12, 33–35]. Besides TNFRs, glial TLRs [3, 5, 36] and inflammasome [4, 5, 37, 38], which are linked to sensing tissue stress/damage, can also promote neuroinflammation in glaucoma. Like TNFR signaling, TLR signaling [12, 14, 39–42] and inflammasome activation [15, 16, 43–46] involve caspase-8 in increased expression, proteolytic activation, and secretion of proinflammatory cytokines. By functioning as a scaffold for RIPK1 recruitment to the signaling complex, procaspase-8 plays an essential protease-independent catalytic role for NF-κB activation and the proinflammatory output [47]. Caspase-8 can also activate various kinases critical for inflammation signaling, such as including RIPK, NIK, and IκKs, and other inflammatory mediators involved in inflammasome-driven processes [11–16]. 

After death receptor engagement, the rapidly activated NF-κB by complex I also enables anti-apoptotic NF-κB targets, including cFLIP, which interfere with the caspase-8 in complex II. As a DED-containing protease-deficient caspase-8 homolog, cFLIP regulates the dynamics of the caspase-8 recruitment to complex II and inhibits apoptosis signaling by preventing its autocleavage-mediated activation. While procaspase-8 presents a high affinity to bind this signaling complex, the recruitment of cFLIP is via heterodimerization with procaspase-8. Due to the high efficiency of caspase-8 binding to complex II, cFLIP mainly prevents the enzymatic activity of caspase-8 rather than its binding to this complex [48, 49]. Evidently, both c-FLIP_L_ and c-FLIP_R_ block caspase-8 activation at different levels of procaspase-8 processing at the complex II [50]. Several models have been reported for the binding of cFLIP isoforms to this complex. According to a recent model, their cooperative hierarchical binding to procaspase-8, depending on the unbound c-FLIP_L_ to cFLIP_S(R)_ ratio, is thought to control enzymatic activities and cell fate [28]. Despite the view that cFLIP requires procaspase-8 for binding to complex II, cFLIP proteins may also act as control checkpoints of DED filament assembly [51]. Apart from TNF receptors, cFLIP also protects by inhibiting the formation of the complex-II-like cell death-inducing platform that other immune receptors, including TLRs, can induce [52]. 

Heterodimerization with cFLIP is known to not only prevent auto-processing of caspase-8 for apoptotic function but also result in a limited and selective substrate specificity initiating diverse signaling outcomes [53–55]. Besides inhibiting caspase-8-mediated apoptosis, an essential role for cFLIP in regulating the life/death decisions is the induction of NF-κB [56–58]. As a caspase-8 substrate for pro-survival functions, procaspase-cleaved DED-containing fragments of cFLIP can induce NF-κB activation via interacting with the IκB kinase (IκK) complex by binding to IκKγ [13, 59, 60]. Thus, besides regulating the functional divergence of caspase-8 in apoptotic and non-apoptotic pathways, cFLIP controls NF-κB activation by directly interacting with multiple proteins upstream of the NF-κB activation pathway. These support cFLIP functions beyond cell death regulation.

The recent study exploring the molecular processes of glaucoma linked cFLIP to cell type-dependent differences in the biological activities of caspase-8, including apoptotic caspase-8 activity in RGCs and the non-apoptotic function of caspase-8 in glial inflammatory responses [20]. As supported by previous studies [61, 62], the high expression level of cFLIP in astroglia was considered a critical factor for their different fate as opposed to RGCs in glaucomatous samples [20]. While the RGCs in glaucomatous samples presented cleaved caspase-8, including the p18 active enzyme, no apoptotic caspase-8 cleavage was detectable in the glaucomatous glia. Instead, astrocytes were detected to express the 55 kDa full-length inactive proenzyme and the partially processed p43 subunit that might result from heterodimerization of procaspase-8 with cFLIP_L_ [50]. 

Cell death and inflammatory signals typically oppose each other downstream of death receptor engagement, as increasing apoptosis may be parallel to reduced inflammation, and caspase inhibition may result in enhanced inflammatory cytokine production. This view is also consistent with the model for caspase-8 activity-dependent cell fate decisions. While the high enzymatic activity of caspase-8 promotes apoptosis in RGCs, in the absence of full proteolytic cleavage, the catalytic activity of caspase-8 signals cell survival and inflammation in astroglia [50]. In the present study, immunoblotting of astroglia proteins detected persisting p55 procaspase with increased caspase-8 cleavage products, including p43 and p18, in *cFLIP*-deleted samples. The detected cleavage products might result from intra- and interdimeric self-cleavage of caspase-8 in the lack of cFLIP [63]. Nevertheless, despite increased self-processing of caspase-8, deletion of *cFLIP* in astroglia could reduce proinflammatory responses in experimental glaucoma while maintaining astroglia survival. Contrasting studies suggest cFLIP functioning as a dual suppressor of apoptosis and inflammation; [64, 65] however, differences in cell type, caspase activity, disease model, and experimental paradigm make the comparisons difficult. As discussed below, additional molecular alterations detected in the current study allow alternative conclusions to be reached.

### Alterations in inflammatory responses of astroglia with *cFLIP* deletion

The molecular outcomes of this study indicated a reduced inflammatory response to ocular hypertension with *cFLIP*-deletion in astroglia. Widespread glial responses during glaucomatous neurodegeneration portray a continuum of multiple activation states exhibiting tissue region- and disease-stage-dependent alterations. A spatial and temporal heterogeneity in glial reactivity embraces a board of morphological and molecular responses. Like previous studies of experimental glaucoma, the present study detected morphological characteristics of reactive astroglia, including hypertrophy of their cellular processes with increased GFAP immunolabeling. However, the quantitative parameters, including the intensity and percentage coverage of GFAP immunolabeling, exhibited only subtle changes between the ocular hypertensive retinas of mice with or without astroglial *cFLIP* or *cFLIP*_*L*_ deletion. Although no significant alteration was detectable in these morphological features, inflammatory responses of the glaucomatous astroglia presented various molecular changes in *GFAP/cFLIP* and *GFAP/cFLIP*_*L*_ mice.

Multiplex immunoassays detected decreased titers of multiple proinflammatory cytokines in the ocular hypertensive *GFAP/cFLIP* and *GFAP/cFLIP*_*L*_ retina and optic nerve compared to ocular hypertensive controls. While proinflammatory cytokines, such as IL1, IL2, IFNγ, and TNFα, presented decreased titers, anti-inflammatory cytokines, including IL4 and IL10, were significantly increased ocular hypertensive tissues with *GFAP/cFLIP* or *GFAP/cFLIP*_*L*_ deletion in astroglia. Similar to the cytokine profiles displayed by immunoassays, the molecular changes detected by NanoString-based analysis of astroglia samples found a significant increase in the anti-inflammatory cytokine response, including *Il10*, and a significant decrease in the proinflammatory cytokines, such as *Il15* and *Il22*, with astroglial *cFLIP* deletion.

As discussed in detail below, the alterations detected in proinflammatory and anti-inflammatory molecules with astroglial *cFLIP* deletion were also parallel to decreased *RelA* but increased *RelB* expression and activity. These molecular outcomes may point to a regulatory mechanism to limit proinflammatory outcomes and prevent cell death.

Additional molecules presenting altered expression in *GFAP/cFLIP* samples included some gene targets of NF-κB. For example, TNFα-induced protein-3 (*Tnfaip3*, also called A20) was among the downregulated molecules in *cFLIP*-deleted astroglia. A20 is known to regulate TNFα-mediated inflammation and apoptosis [66–68]. The decreased expression of Tnfaip3, which was previously found to be an epigenetic target in glaucomatous human eyes [4], might be secondary to inhibited TNFR signaling and inflammatory outcomes.

In addition, *cFLIP*-deleted astroglia upregulated several complement components. Previous studies have demonstrated the complement system as a potent modulator and effector of diverse glial functions in glaucomatous neurodegeneration [27, 69]. Astrocytes normally maintaining the homeostasis of the central nervous system are well-equipped to receive and send complement-related signals. Many of the molecules involved in the complement activation pathway, including various receptors and regulators, are expressed by astrocytes. They play critical roles in their crosstalk with microglia, besides tissue clearance, protection, or damage when dysregulated. Evidently, the dynamic shift between different activation states and functions of astrocytes in the early or late stages of glaucoma includes complement components [70]. For example, transgenic targeting of complement C3 delayed the progression of neurodegeneration in mice with hereditary glaucoma; [71] however, C3 induced neuroprotective functions of astrocytes at early disease stages [72]. 

### Alterations in NF-κB expression of astroglia with *cFLIP* deletion

A remarkable observation from the NanoString-based molecular analysis was decreased *RelA* but increased *RelB* expression in *cFLIP*-deleted astroglia. This observation was also matched with decreased phospho-RelA and increased phospho-RelB expression in *cFLIP-*deleted astroglia. The five structurally related monomers of the NF-κB signaling system, including RelA (p65), RelB, NF-κB1 (p105/p50), NF-κB2 (p100/p52), and c-Rel, mediates dimerization, interaction with IκBs, and nuclear translocation for transcription of several inflammatory, developmental, and survival genes. Various hetero- or homodimers mediate transcription of target genes by binding to a specific DNA element, κB enhancer. Different NF-κB dimeric complexes are expressed in a cell type- and stimulus-specific manner. NF-κB family members present sequence homology in their DNA binding and dimerization domains and participate in transcriptional activation of hundreds of genes; however, despite their close relation in sequence and function, RelA and RelB also display distinct effects on gene expression [73, 74]. Multiple studies have demonstrated that they modulate each other’s function and repress NF-κB-dependent gene expression [75, 76]. Target genes for the transcription mediated through RelA: p50 and cRel: p50 heterodimers in the canonical pathway are mainly involved in innate immunity. Although inflammatory responses typically occur through activation of the canonical pathway, RelB, with its DNA-binding partners p50 and p52, plays its specific roles along the non-canonical NF-κB pathway. The new synthesis of RelB allows for the generation of RelB: p52 in this alternative pathway, which is insensitive to IκB control. The non-canonical NF-κB pathway appears to be a supplementary signaling axis that cooperates with the canonical pathway and governs the gene regulation predominantly involved in lymphoid organ development and adaptive immunity [77–80]. Previous studies have shown cross-regulation between RelA and RelB on TNFα-induced NF-κB target gene expression. RelA regulates the transcription of RelB [81], promotes the generation of RelB/p52 dimers [82], and plays a regulatory role to diminish RelB activity for selective target gene expression in response to TNFα [76]. A more recent study supporting a cross-talk between the canonical and non-canonical NF-κB pathways showed that reduction in cellular RelB levels led to dysregulation of the NF-κB pathway, promoting a shift towards proinflammation due to the removal of the RelB-mediated inhibition of RelA-induced proinflammatory transcriptional activity [83]. 

Unlike RelA, RelB remained understudied in glaucoma research. This is partly because TNFα signaling, a prominent component of the astroglia-driven neuroinflammation in the glaucomatous retina, appears to mainly induce RelA: p50 dimers [84]. Interestingly, molecular analysis detected decreased *RelA* expression and phosphorylated RelA with *cFLIP*-deletion in ocular hypertensive astroglia. On the contrary, *RelB* expression and phospho-RelB were increased, supporting an upregulated transcriptional function of RelB [85, 86] in ocular hypertensive *GFAP/cFLIP* compared to ocular hypertensive controls. These findings indicate the non-canonical pathway activated in ocular hypertensive samples of *cFLIP*-deleted astroglia. Since the canonical and non-canonical NF-κB pathways may impact each other, we wonder whether the increased *RelB* expression we detected could be compensatory to decreased *RelA* or an independent response for further repressing the proinflammatory activation. In support of this view, *RelB* knock-out mice were reported to suffer multi-organ inflammation [87], and overexpression of *RelB* in LPS-stimulated macrophages suppressed proinflammatory TNFα production [88]. In more recent studies, increased expression of *RelB* in astrocytes induced immune tolerance in experimental neuroinflammation due to silencing the proinflammatory cytokine genes after RelB: p50 binding to DNA [89, 90]. 

Thus, the molecular data pointed to a regulatory mechanism to diminish *RelA* and promote *RelB* for selective expression of NF-κB target genes in *cFLIP*-deleted astroglia. This molecular regulation seems to limit the expression of a subset of RelA-responsive genes, including proinflammatory cytokines. Respecting the cell type-specific differences in NF-κB activation and function [91], a better understanding of the cell type-specificity of these observations awaits further investigation. As discussed below, the changed expression pattern of *RelA* and *RelB* may also have implications for astroglia survival of the *cFLIP* deletion.

### Transgenic effects on survival of astroglia with *cFLIP* deletion

**Fig. 9:**
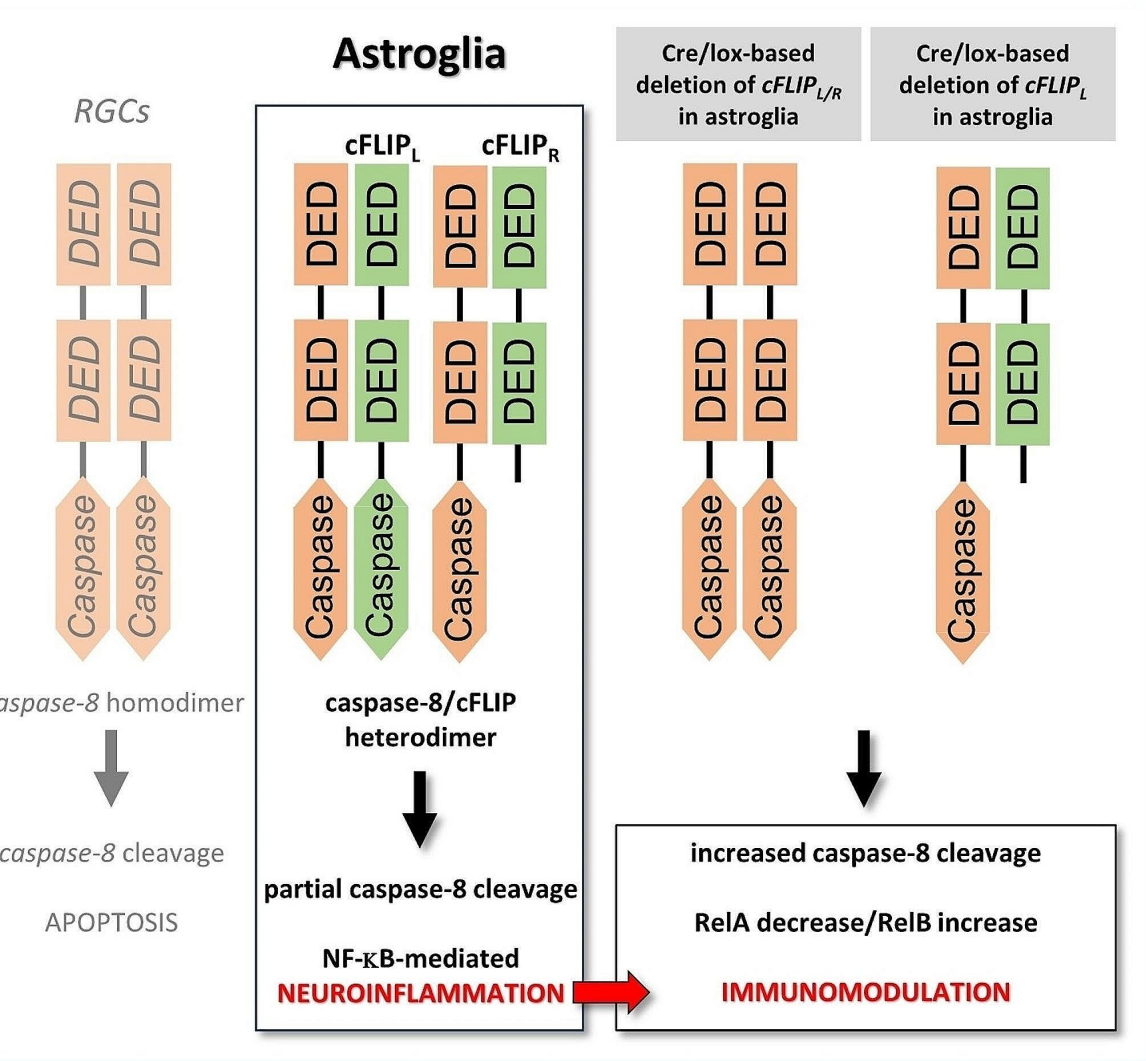
Modeling of the experimental outcomes. While the longer isoform of cFLIP (cFLIP_L_) is composed of tandem death effector domains (DEDs) and a catalytically inactive caspase-like domain, the short isoform (c-FLIP_R_) is a truncated version. As a protease-deficient caspase-8 homolog, the heterodimer of cFLIP with caspase-8 prevents autocleavage-mediated apoptotic activation requiring homodimerization (as detected in glaucomatous RGCs). Besides inhibiting caspase-8-mediated apoptosis, high cFLIP expression also plays a critical role in regulating NF-κB-mediated inflammatory outcomes (as detected in glaucomatous astroglia). The findings of this study support that deletion of *cFLIP*, or *cFLIP*_*L*_, in astroglia can reduce proinflammatory responses and inflammatory injury to RGCs in experimental glaucoma. Accompanying molecular alterations also imply that a transcriptional autoregulatory response, including induction of RelB, reinforces pro-survival, despite increased caspase-8 cleavage in *cFLIP*-deleted astroglia

Concerning the critical role of caspase-8/cFLIP interactions in cell fate, one possibility after *cFLIP* deletion could be the signal shift toward caspase-8-initiated apoptosis. Although caspase-8:cFLIP_L_ heterodimers may possess restricted protease activity, and the resultant cleavage product, p43, can cleave some downstream effectors of apoptosis, short isoform can terminate the formation of tandem homo-oligomers propagating cleavage-mediated caspase-8 activation [28]. As modeled in Fig. 9, in the mice with astroglial *cFLIP*_*L*_ deletion, intact *cFLIP*_*R*_ (that does not have a caspase-like domain, but DED, common for both isoforms) can prevent procaspase-8 homo-oligomer assembly needed for the full cleavage. This study detected increased caspase-8 cleavage; however, no change was detectable when apoptotic cell death was analyzed in astroglia. Not only *GFAP/cFLIP*_*L*_ but also *GFAP/cFLIP*, lacking both isoforms, presented no induction of astroglial apoptosis at least at 12 weeks of ocular hypertension.

There may be different explanations for protection against apoptosis, which warrants additional studies to elucidate further. Most importantly, the molecular alterations detected seemed decisive for cell survival. While decreased *RelA* might be associated with reduced inflammatory response, increased *RelB* might be sufficient to prevent apoptosis since it is known to promote several anti-apoptotic gene targets [91, 92]. Previous *RelB* mutant studies demonstrated the contribution of RelB to anti-apoptotic signaling and cell survival during thymocyte maturation [93]. The anti-apoptotic gene expression might persist despite RelB-mediated silencing of the cytokine response [76, 94]. Studies using *RelB*-deficient cells showed that the selective target gene expression by RelB includes an abundance of anti-apoptotic molecules, including Bcl-2 and Bcl-xL, and inhibitor of apoptosis proteins (IAP), in addition to the regulators of cell cycle transition and proliferation, such as c-Myc and cyclins [95, 96]. Besides inhibiting cell death, the IAP controls ubiquitin-dependent pathways that regulate RIPK1/NF-κB activation, thereby modulating inflammation [97]. Moreover, RelB might contribute to cellular protection by inducing an antioxidant response [98, 99]. The decreased *nos2* detected in *cFLIP*-deleted astroglia seems consistent with such an antioxidant response.

Additionally, caspase-8 auto-processing through a combined series of intra- and interdimeric cleavages is accelerated with increasing ligand binding for receptor signaling [97]. Therefore, decreased production of specific proapoptotic ligands, such as proinflammatory cytokine TNFα, and decreased stimulus strength of the death receptor might cause weakness in receptor signaling, contributing to the control of apoptosis. In similar interest to RelB, *A20*, also decreased in expression in *cFLIP*-deleted astroglia, is known to suppress both classical and alternative NF-κB and block the expression of specific anti-apoptotic genes [98].

Thus, the immunomodulatory transgenic effect with astroglial *cFLIP* deletion did not shift the experimental paradigm to caspase-8-mediated cell death; however, a transcriptional autoregulatory response, including induction of *RelB*, was considered to reinforce pro-survival. Ongoing studies through a more chronic period of ocular hypertension are expected to provide further information to determine whether cell survival in *cFLIP*-deleted astroglia endures long-term. It should also be clarified whether apoptosis, if induced long-term, would specifically affect astroglia’s proinflammatory/neurotoxic states because these cells would be primed for caspase-8 expression and activities through TNFR, TLR signaling, or the inflammasome activation in glaucoma.

## Conclusions

In summary, the findings of this study accumulated supportive data for the astroglial *cFLIP* deletion to prevent/reverse the neurodegenerative proinflammatory outcomes of experimental glaucoma. The transgenic modulation of *cFLIP* expression in astroglia activated a transcriptional autoregulatory response, dampening RelA but boosting RelB for selective expression of NF-κB target genes, reinforcing cell survival in *cFLIP*-deleted astroglia while limiting proinflammatory responses. In light of the presented findings, targeting the caspase-8/cFLIP interaction in astroglia may offer a treatment strategy against NF-κB-regulated neuroinflammation and inflammatory injury to neurons. Concerning the direct cytotoxicity of astroglia-produced proinflammatory cytokines, including TNFα [8–10], to RGCs in glaucoma and a prolonged amplification cycle of the glia-driven neurodegenerative inflammation stimulated by downstream NF-κB activation [6], such an astroglia-targeting immunomodulatory approach can provide long-term protection to these precious neurons. Continuing studies for improved molecular understanding of neuroinflammation will be the key to opening the way to a therapeutic approach by immunomodulation in glaucoma and other neuroinflammatory neurodegenerative diseases.

### Electronic supplementary material

Below is the link to the electronic supplementary material.

Supplementary Material 1

## Data Availability

The generated and analyzed data during this study are included in this article, and methodological details and materials will be available to interested parties upon request.

## Abbreviations

Cflar: Caspase-8 FADD-like apoptosis regulator
cFLIP: FLICE-like inhibitory protein
DED: Death effector domain
GFAP: Glial fibrillary acidic protein
IκKβ: Inhibitor of kappaB kinase-subunit beta
IFN-γ: Interferon-gamma
IL: Interleukin, NF-κB, NIK, NF-κB-inducing kinase, nuclear factor-kappaB
RELA/B: v-Rel avian reticuloendotheliosis viral oncogene homolog A/B
RIPK: Receptor-interacting serine/threonine-protein kinase 1,RGC, retinal ganglion cell
RPBMS: RNA-binding protein with multiple splicing
TLR: toll-like receptor
TNF-α: Tumor necrosis factor-alpha
TNFR: Tumor necrosis factor receptor
TUNEL: Terminal deoxynucleotidyl transferase (TdT) dUTP Nick-End Labeling
