# Supplementary figures and images for: cFLIP in the molecular regulation of astroglia-driven neuroinflammation in experimental glaucoma
Source: J Neuroinflammation. 2024 Jun 1;21:145. doi: 10.1186/s12974-024-03141-4 (PMC11143607; doi:10.1186/s12974-024-03141-4)

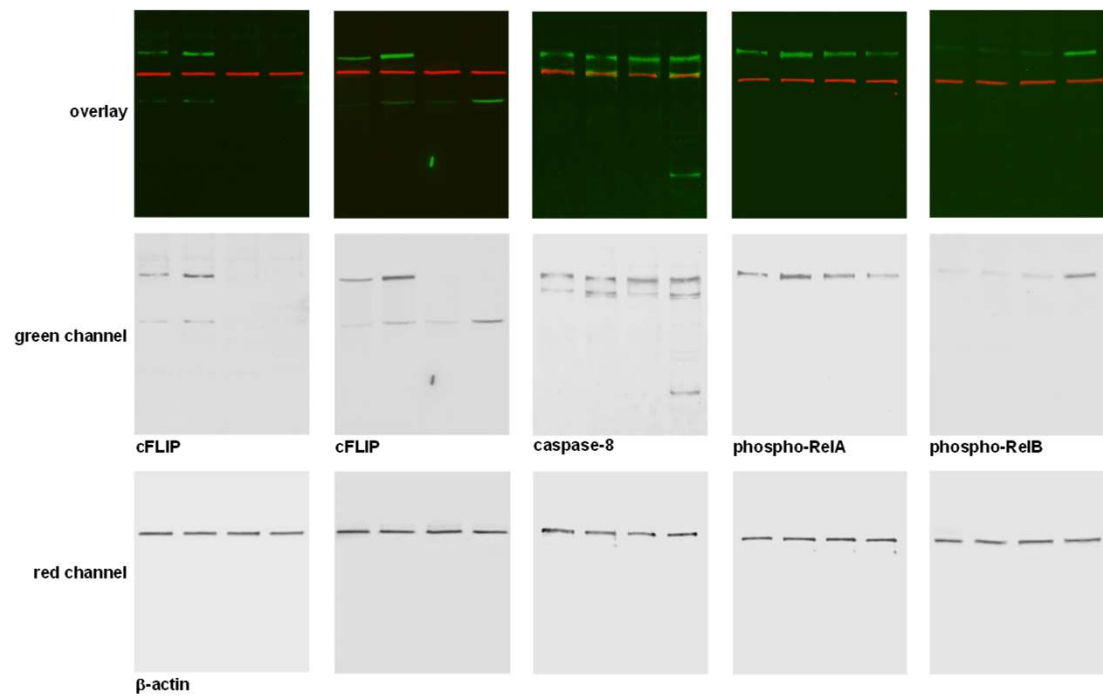

Supplement: Supplementary file 1 — Supplementary Material 1 [file 12974_2024_3141_MOESM1_ESM.pdf]
